# Epigenetic activation of the small GTPase TCL contributes to colorectal cancer cell migration and invasion

**DOI:** 10.1038/s41389-020-00269-9

**Published:** 2020-09-30

**Authors:** Baoyu Chen, Zhiwen Fan, Lina Sun, Junliang Chen, Yifei Feng, Xiangshan Fan, Yong Xu

**Affiliations:** 1 Key Laboratory of Targeted Invention of Cardiovascular Disease and Collaborative Innovation Center for Cardiovascular Translational Medicine, Department of Pathophysiology, Nanjing Medical University, Nanjing, China; 2 Department of Pathology, Affiliated Nanjing Drum Tower Hospital of Nanjing University School of Medicine, Nanjing, China; 3 Institute of Biomedical Research, Liaocheng University, Liaocheng, China; 4 Department of Pathology and Pathophysiology, School of Biological and Basic Medical Sciences, Soochow University, Soochow, China; 5 Department of Pathophysiology, Wuxi Medical School, Jiangnan University, Wuxi, China; 6 Department of Colorectal Surgery, First Affiliated Hospital of Nanjing Medical University and The First School of Clinical Medicine, Nanjing Medical University, Nanjing, China

**Keywords:** Epigenetics, Histone post-translational modifications, Metastasis, Cell migration

## Abstract

TC10-like (TCL) is a small GTPase that has been implicated in carcinogenesis. Elevated TCL expression has been observed in many different types of cancers although the underlying epigenetic mechanism is poorly understood. Here we report that TCL up-regulation was associated with high malignancy in both human colorectal cancer biopsy specimens and in cultured colorectal cancer cells. Hypoxia, a pro-metastatic stimulus, up-regulated TCL expression in HT-29 cells. Further studies revealed that myocardin-related transcription factor A (MRTF-A) promoted migration and invasion of HT-29 cells in a TCL-dependent manner. MRTF-A directly bound to the proximal TCL promoter in response to hypoxia to activate TCL transcription. Chromatin immunoprecipitation (ChIP) assay showed that hypoxia stimulation specifically enhanced acetylation of histone H4K16 surrounding the TCL promoter, which was abolished by MRTF-A depletion or inhibition. Mechanistically, MRTF-A interacted with and recruited the H4K16 acetyltransferase hMOF to the TCL promoter to cooperatively regulate TCL transcription. hMOF depletion or inhibition attenuated hypoxia-induced TCL expression and migration/invasion of HT-29 cells. In conclusion, our data identify a novel MRTF-A-hMOF-TCL axis that contributes to colorectal cancer metastasis.

## Introduction

Colorectal carcinoma (CRC) represents one of the leading causes for cancer-related deaths worldwide^1^. With over 1 million new cases each year, CRC is the third most commonly diagnosed malignancy globally. Despite the development of sophisticated screening techniques that detect early polyps and the use of chemotherapeutic drugs to treat patients under the guidelines of precision medicine, CRC remains the third most lethal form of cancers to which close to 700,000 patients succumb yearly^2^. The prognosis for those with late-stage and highly malignant CRC is especially dim due to widespread metastasis^3^. These highly malignant types of CRC are characterized by aggressive migratory and invasive behaviors. A wide range of pro-metastatic stimuli can promote migration and invasion of CRC cells. For instance, hyper-proliferation of cancer cells limits the availability of oxygen supply to create a hypoxic micro-environment. Hypoxia in turn promotes cancer cell migration and invasion via multiple different mechanisms. Hypoxia can stimulate epithelial-mesenchymal transition (EMT) of CRC cells, a process in which cancer cells discard epithelial markers and acquire mesenchymal-like characteristics thus becoming more capable of migration and invasion^4–6^. Hypoxia can also induce the expression and release of metalloproteinases (MMPs) from CRC cells to remodel extracellular matrix and facilitate migration/invasion^7^. In addition, hypoxia, via hypoxia-inducible factor 1 (HIF-1α), enhances the canonical Wnt signaling to sustain the stemness of CRC cells and greatly augment migration/invasion^8^. Another potential mechanism by which hypoxia promotes CRC malignancy is suggested by the observation that PGC-1a, up-regulated by low oxygen content, regulates mitochondrial function to fuel the aberrant migration and invasion of CRC cells.

TC10-like (TCL), also known as RhoJ, is a member of the small GTPase superfamily that shares significant homology with Cdc42^9^. Early characterization of its function revealed that TCL plays a crucial role in adipogenesis likely by activating PPARγ^10^ and by regulating mitotic clonal expansion^11^. More recently, it has been demonstrated by independent investigations that TCL may regulate cell mobility. For instance, Heath and colleagues have observed that TCL is located to the focal adhesions in endothelial cells^12^. Pro-angiogenic stimuli activates TCL, which in turn down-regulates formation of stress fibers and actomyosin contractility to facilitate cell migration. Mechanistically, TCL interacts with the scaffolding protein GIT–PIX to disassemble focal adhesion allowing the cells to move forward^13^. The observation that TCL activity is positively associated with cell motility has since been confirmed in cornea epithelial cells^14^ and cancer cells^15,16^.

Mounting evidence suggests that TCL expression is elevated and correlated with poor prognosis in patients with malignant types of cancers^15–18^. We have previously reported that TCL mediates hypoxia-induced endothelial-mesenchymal transition by activating the H3K4 methyltransferase WDR5^19^. It remains unknown whether TCL expression levels are correlated with CRC malignancy and, if so, how TCL expression is regulated. Here we provide evidence to show that the histone acetyltransferase hMOF, recruited by the transcriptional modulator MRTF-A, mediates hypoxia-induced TCL expression to promote CRC cell migration and invasion. Therefore, targeting this novel MRTF-A-hMOF-TCL axis may yield novel therapeutic solutions against colorectal cancer metastasis.

## Results

### High TCL expression correlates with CRC malignancy

**Fig. 1:**
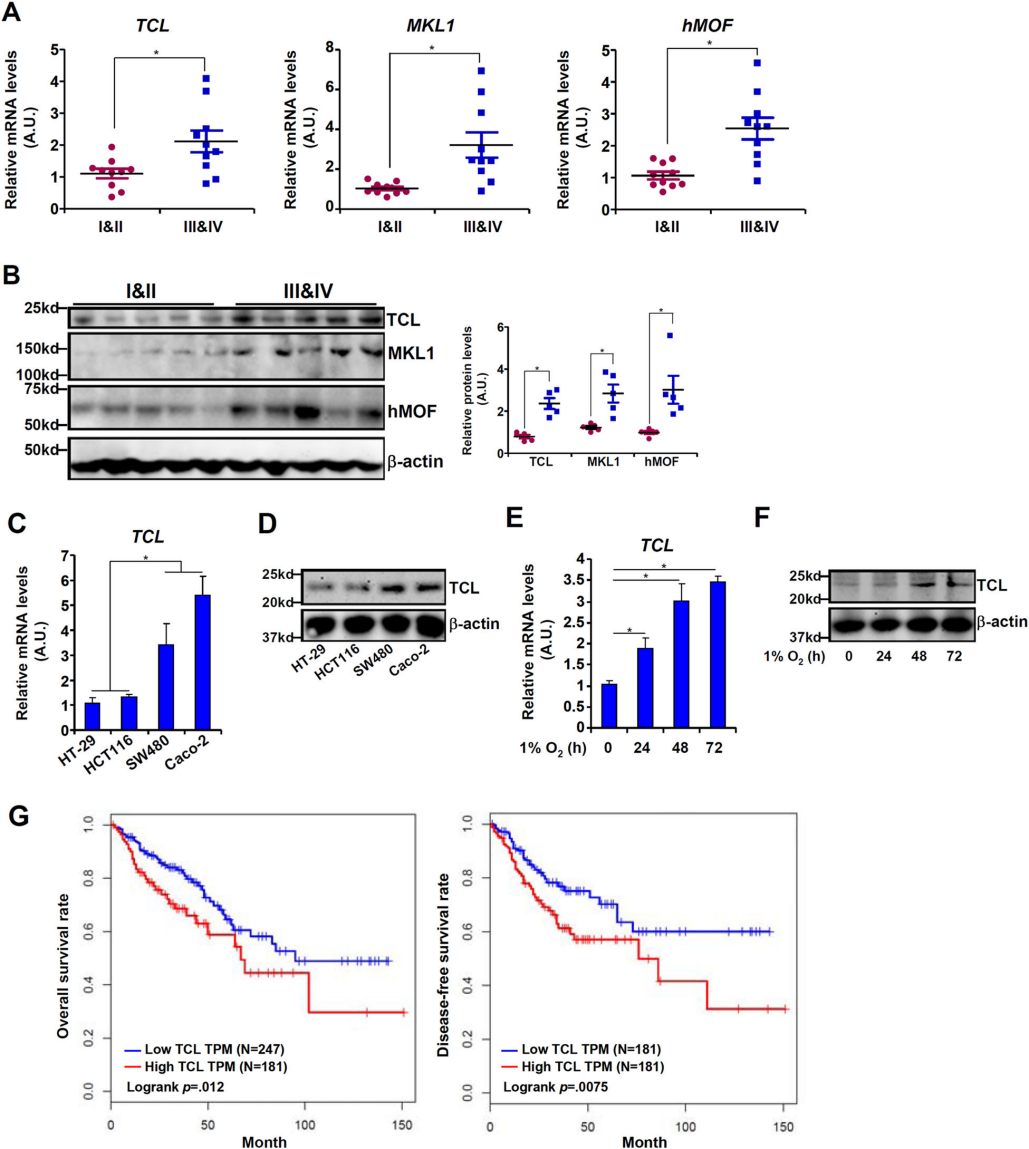
High TCL expression correlates with CRC malignancy. **a**, **b** Gene expression levels in human CRC specimens are examined by qPCR and western blotting. **c**, **d** TCL expression levels in a panel of CRC cells were examined by qPCR and western. **e**, **f** HT-29 cells were exposed to 1% O_2_ and harvested at indicated time points. TCL expression levels were examined by qPCR and western. Data represent averages of three independent experiments and error bars represent SEM. **g** Kaplan–Meier plot of survival in patients with high and low TCL expression.

We first evaluated the correlation between TCL expression levels and colorectal cancer malignancies in a small cohort of patients. As shown in Fig. 1a, b, TCL expression was significantly up-regulated as CRC progressed from a low-malignant type (grade I&II) to a high-malignant type (grade III&IV). We also compared TCL expression levels in a panel of CRC cell lines with varying metastatic abilities. TCL expression, at both mRNA (Fig. 1c) and protein (Fig. 1d) levels, were much higher in SW480 cells and Caco-2 cells than in HT-29 cells and HCT116 cells. Exposure to hypoxia, a well-established pro-metastatic stimulus, significantly up-regulated TCL expression in HT-29 cells (Fig. 1e, f) and HCT116 (Fig. S1) cells. More important, Kaplan–Meier analysis using data from the TCGA database with the help of a bioinformatics web server (http://gepia.cancer-pku.cn) revealed that high TCL expression was associated with poorer disease-free survival in patients with CRC (Fig. 1g). Together, these data suggest that high TCL expression may be correlated with CRC malignancy.

### MRTF-A contributes to CRC cell migration and invasion by regulating TCL expression

**Fig. 2:**
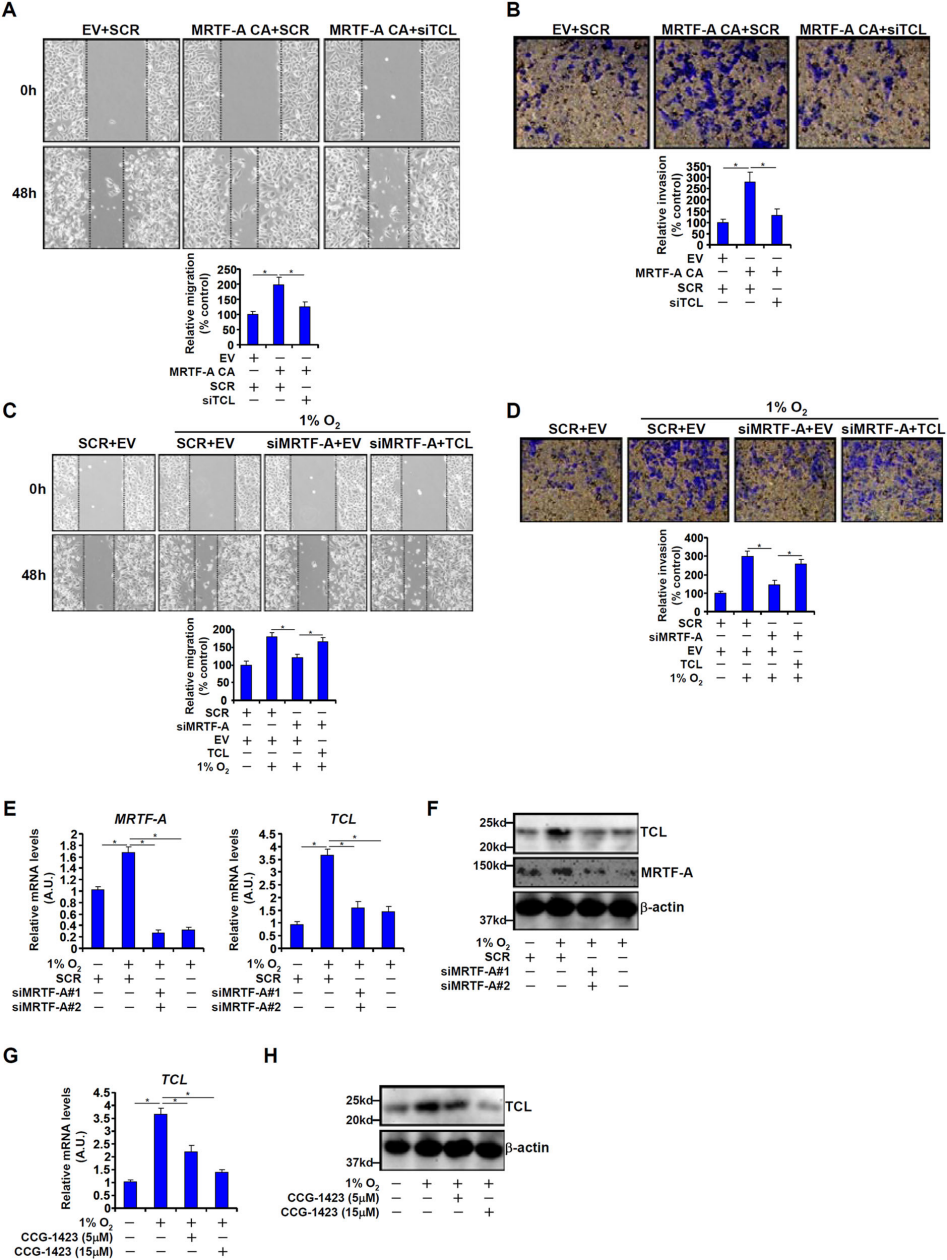
MRTF-A contributes to CRC cell migration and invasion by regulating TCL expression. **a** A constitutively active (CA) MRTF-A was transfected into HT-29 cells with or without siRNA targeting TCL. Cell migration was measured by scratch wound healing assay and quantified by Image Pro as described in Methods. **b** A constitutively active (CA) MRTF-A was transfected into HT-29 cells with or without siRNA targeting TCL. Cell migration was measured by transwell assay and quantified by Image Pro as described in Methods. **c** HT-29 cells were transfected with siRNA targeting MRTF-A or scrambled siRNA (SCR) in the presence or absence of TCL followed by treatment with 1% O_2_ for 48 h. Cell migration was measured by scratch wound healing assay and quantified by Image Pro as described in Methods. **d** HT-29 cells were transfected with siRNA targeting MRTF-A or scrambled siRNA (SCR) in the presence or absence of TCL followed by treatment with 1% O_2_ for 48 h. Cell invasion was measured by transwell assay and quantified by Image Pro as described in Methods. **e**, **f** HT-29 cells were transfected with siRNA targeting MRTF-A or scrambled siRNA (SCR) followed by treatment with 1% O_2_ for 48 h. Gene expression levels were examined by qPCR and western. **g**, **h** HT-29 cells were treated with 1% O_2_ in the presence or absence of CCG-1423 for 48 h. Gene expression levels were examined by qPCR and western. Data represent averages of three independent experiments and error bars represent SEM.

Myocardin-related transcription factor A (MRTF-A) is a transcriptional modulator that has been implicated in carcinogenesis. Similar to TCL, MRTF-A levels were also higher in more malignant forms of human CRC specimens (Fig. 1b). Over-expression of a constitutively active (CA) form of MRTF-A promoted the migration and invasion of TCL-low HT-29 cells (Fig. 2a, b) and HCT116 cells (Fig. S2A, B). Of interest, depletion of endogenous TCL blocked the augmentation of cell migration/invasion by MRTF-A. In addition, MRTF-A knockdown weakened migration/invasion of HT-29 cells (Fig. 2c, d) and HCT116 cells (Fig. S2C, D) exposed to hypoxia stimulation whereas forced expression of exogenous TCL restored cell migration/invasion. On the contrary, depletion of MRTF-A or TCL with siRNAs similarly attenuated the migration (Fig. S3A) and invasion (Fig. S3B) of SW480 cells and Caco-2 cells.

These data suggest that MRTF-A may contribute to CRC cell migration and invasion by regulating TCL expression. In support of this notion, MRTF-A depletion by two separate pairs of siRNAs markedly suppressed TCL induction by hypoxia in HT-29 cells (Fig. 2e, f) and HCT116 cells (Fig. S2E, F). In the TCL-high SW480 (Fig. S3C, D) and Caco-2 (Fig. S3E, F) cells, MRTF-A knockdown similarly repressed basal TCL expression. Further, treatment with CCG-1423, a small-molecule inhibitor of MRTF-A, also attenuated TCL induction by hypoxia (Fig. 2g, h).

### MRTF-A directly regulates TCL transcription

**Fig. 3:**
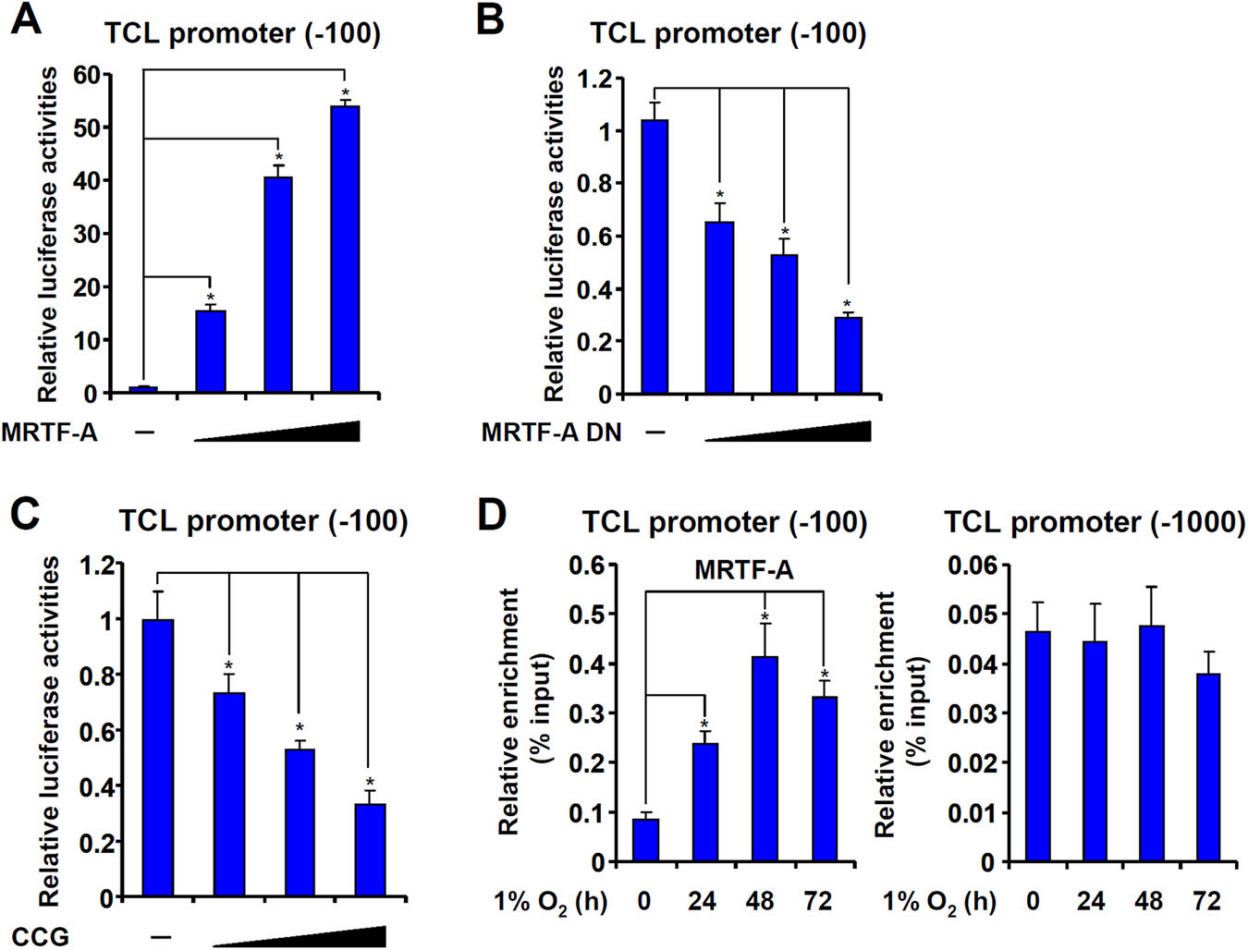
MRTF-A directly regulates TCL transcription. **a** HEK293 cells were transfected with a TCL promoter-luciferase construct and increasing doses of MRTF-A. Luciferase activities were normalized by protein concentration and GFP fluorescence and expressed as relative luciferase unit compared to the control group. **b** HEK293 cells were transfected with a TCL promoter-luciferase construct and increasing doses of dominant-negative (DN) MRTF-A. Luciferase activities were normalized by protein concentration and GFP fluorescence and expressed as relative luciferase unit compared to the control group. **c** HEK293 cells were transfected with a TCL promoter-luciferase construct and increasing doses of CCG-1423. Luciferase activities were normalized by protein concentration and GFP fluorescence and expressed as relative luciferase unit compared to the control group. **d** HT-29 cells were exposed to 1% O_2_ and harvested at indicated time points. ChIP assays were performed with anti-MRTF-A or IgG. Data represent averages of three independent experiments and error bars represent SEM.

Next, we assessed the possibility that MRTF-A may regulate TCL expression at the transcription level. To this end, a luciferase reporter gene fused to the minimal TCL promoter^20^ (−100/+142) was transfected into HEK293 cells. Over-expression of MRTF-A dose-dependently activated the TCL promoter (Fig. 3a). On the contrary, a dominant-negative MRTF-A that lacks the trans-activation domain^21^ repressed the TCL promoter (Fig. 3b). In keeping with these observations, inhibition of MRTF-A activity by CCG-1423 also down-regulated the TCL promoter activity (Fig. 3c). Chromatin immunoprecipitation (ChIP) assay confirmed that hypoxia stimulation promoted the binding of MRTF-A to the proximal TCL promoter (−132/+25) but not the distal TCL promoter (−1018/−872), which contains no known TF binding motifs, in HT-29 cells (Fig. 3d). These data combined suggest that MRTF-A may directly bind to the TCL promoter to activate transcription.

### MRTF-A modulates histone H4K16 acetylation surrounding the TCL promoter

**Fig. 4:**
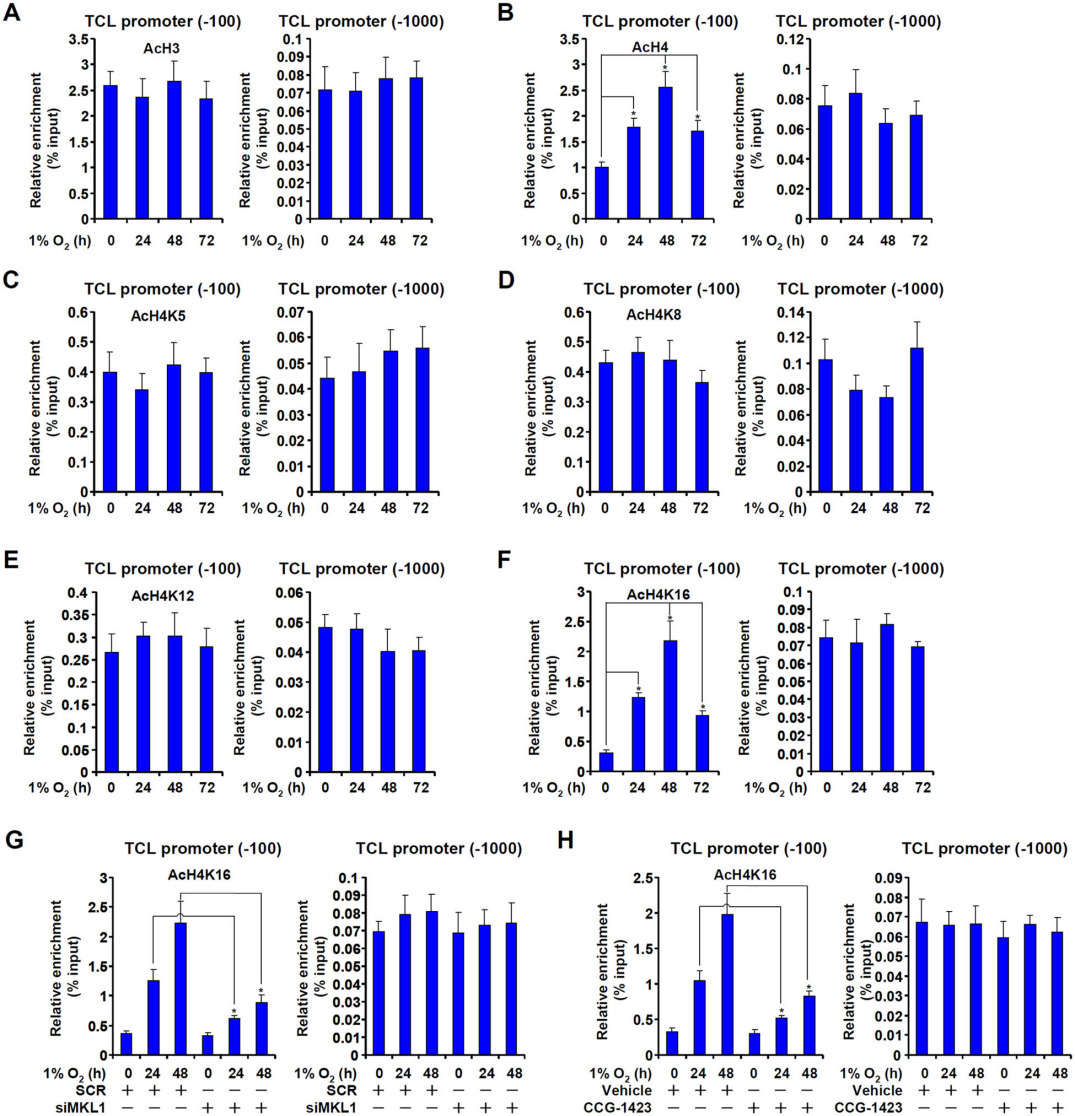
MRTF-A modulates histone H4K16 acetylation surrounding the TCL promoter. **a**–**f** HT-29 cells were exposed to 1% O_2_ and harvested at indicated time points. ChIP assays were performed with anti-acetyl H3 (**a**), anti-acetyl H4 (**b**), anti-acetyl H4K5 (**c**), anti-acetyl H4K8 (**d**), anti-acetyl H4K12 (**e**), and anti-acetyl H4K16 (**f**). **g** HT-29 cells were transfected with siRNA targeting MRTF-A or scrambled siRNA (SCR) followed by treatment with 1% O_2_ for 48 h. ChIP assays were performed with anti-acetyl H4K16. **h** HT-29 cells were treated with 1% O_2_ in the presence or absence of CCG-1423 for 48 h. ChIP assays were performed with anti-acetyl H4K16. Data represent averages of three independent experiments and error bars represent SEM.

When HT-29 cells were exposed to hypoxia, there were changes in histone acetylation surrounding the TCL promoter. Abundant H3 acetylation was already associated with the proximal TCL promoter under normoxic conditions and hypoxia did not alter H3 acetylation (Fig. 4a). On the contrary, H4 acetylation was significantly up-regulated by hypoxia stimulation surrounding the proximal, but not the distal, TCL promoter (Fig. 4b). Further analyses revealed that among the four major lysines within the H4 tail, acetylation levels of K5 (Fig. 4c), K8 (Fig. 4d), and K12 (Fig. 4e) were not appreciably changed by hypoxia surrounding the TCL promoter. H4K16 acetylation, on the other hand, was significantly higher after than before hypoxia exposure (Fig. 4f). Both MRTF-A knockdown by siRNAs (Fig. 4g) and MRTF-A inhibition (Fig. 4h) led to diminished accumulation of H4K16 acetylation surrounding the TCL promoter. These data suggest that H4K16 acetylation may be the rate-limiting step in hypoxia-induced TCL transcription and that MRTF-A may play an essential role in this process.

### MRTF-A interacts with hMOF to regulate TCL transcription in CRC cells

**Fig. 5:**
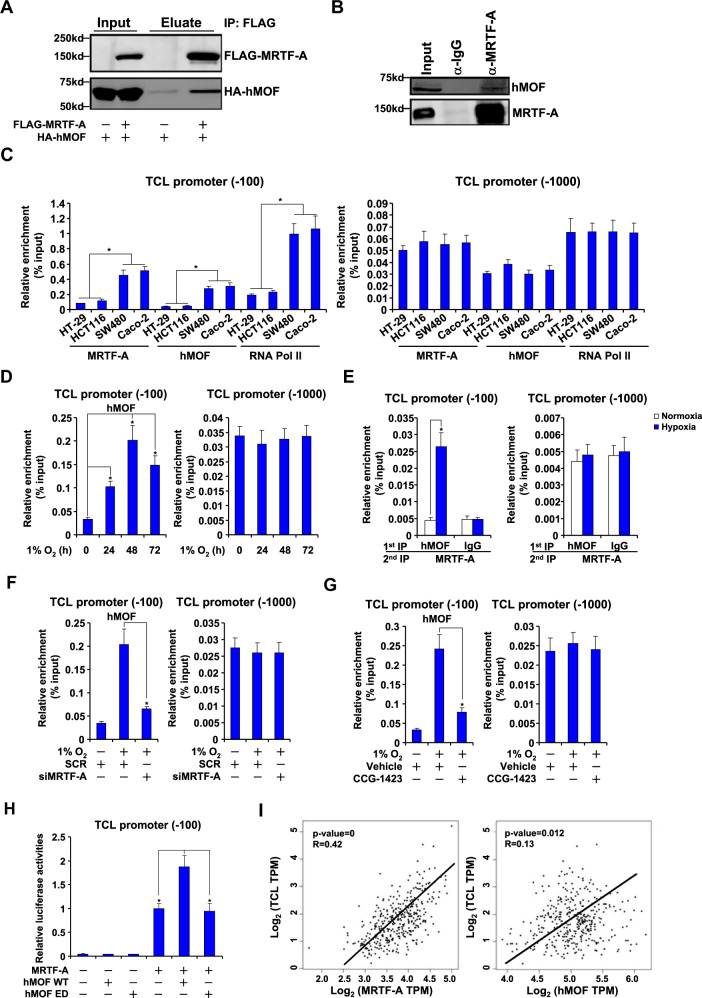
MRTF-A interacts with hMOF to regulate TCL transcription in CRC cells. **a** HEK293 cells were transfected with indicated expression constructs. Immunoprecipitation was performed with anti-FLAG or anti-HA. **b** Nuclear lysates were extracted from HT-29 cells and immunoprecipitation was performed with indicated antibodies. **c** Nuclear lysates were extracted from a panel of different cells and ChIP assay was performed with anti-MRTF-A or anti-hMOF. **d** HT-29 cells were exposed to 1% O_2_ and harvested at indicated time points. ChIP assay was performed with indicated antibodies. **e** HT-29 cells were exposed to 1% O_2_ for 48 h. Re-ChIP assay was performed with indicated antibodies. **f** HT-29 cells were transfected with siRNA targeting MRTF-A or scrambled siRNA (SCR) followed by treatment with 1% O_2_ for 48 h. ChIP assay was performed with anti-hMOF. **g** HT-29 cells were treated with 1% O_2_ in the presence or absence of CCG-1423 for 48 h. ChIP assay was performed with anti-hMOF. **h** HEK293 cells were transfected with a TCL promoter-luciferase construct, MRTF-A, and/or hMOF. Luciferase activities were normalized by protein concentration and GFP fluorescence and expressed as relative luciferase unit compared to the control group. Data represent averages of three independent experiments and error bars represent SEM. **i** Expression data of MRTF-A, hMOF and TCL were extracted from the TCGA database to draw the scatter plot. Pearson correlation co-efficient was calculated.

hMOF is the dedicated H4K16 acetyltransferase in mammals. Based on the observation that MRTF-A deficiency was associated with reduced H4K16 acetylation surrounding the TCL promoter, we hypothesized that an interplay between MRTF-A and hMOF may be accountable. Several lines of evidence supported this hypothesis. First, when exogenous FLAG-MRTF-A and HA-hMOF were over-expressed in HEK293 cells, co-immunoprecipitation assay showed that an anti-FLAG antibody precipitated both MRTF-A and hMOF (Fig. 5a). In addition, co-immunoprecipitation assay also showed that endogenous MRTF-A and hMOF interacted with each other in HT-29 cells (Fig. 5b). Second, ChIP assay showed that MRTF-A bound to the TCL promoter with higher affinity in SW480 cells and Caco-2 cells than in HT-29 cells and HCT116 cells; hMOF displayed a similar binding pattern as MRTF-A (Fig. 5c). Of interest, higher occupancies of RNA polymerase II (Pol II) were detected in SW480 cells and Caco-2 cells than in HT-29 cells and HCT116 cells (Fig. 5c), confirming altered expression of TCL in these cells likely stems from differential transcription rate. In addition, hypoxia exposure stimulated the recruitment of Pol II to the TCL promoter in HT-29 cells (Fig. S4). MRTF-A knockdown suppressed the binding of RNA Pol II to the TCL promoter in SW480 cells and Caco-2 cells (Fig. S5) and in hypoxia-exposed HT-29 cells (Fig. S4) suggesting that MRTF-A may contribute to the regulation of TCL expression by influencing RNA polymerase II access to the promoter and transcription initiation.

Hypoxia treatment promoted the binding of hMOF on the TCL promoter in HT-29 cells with a similar kinetics as MRTF-A (Fig. 5d). More importantly, hypoxia enhanced the interaction between MRTF-A and hMOF on the TCL promoter in HT-29 cells (Fig. 5e) and HCT116 cells (Fig. S6). Third, either MRTF-A depletion (Fig. 5f) or MRTF-A inhibition (Fig. 5g) abrogated hMOF binding to the TCL promoter induced by hypoxia. Fourth, co-expression of hMOF with MRTF-A activated the TCL promoter more strongly than over-expression of MRTF-A alone (Fig. 5h). Finally, using data extracted from the TCGA database, a positive correlation was detected between TCL and MRTF-A or hMOF in patients with colorectal cancer (Fig. 5i). Combined, these data suggest that MRTF-A may rely on hMOF to regulate TCL transcription in CRC cells.

### hMOF contributes to CRC malignancy by regulating TCL transcription

**Fig. 6:**
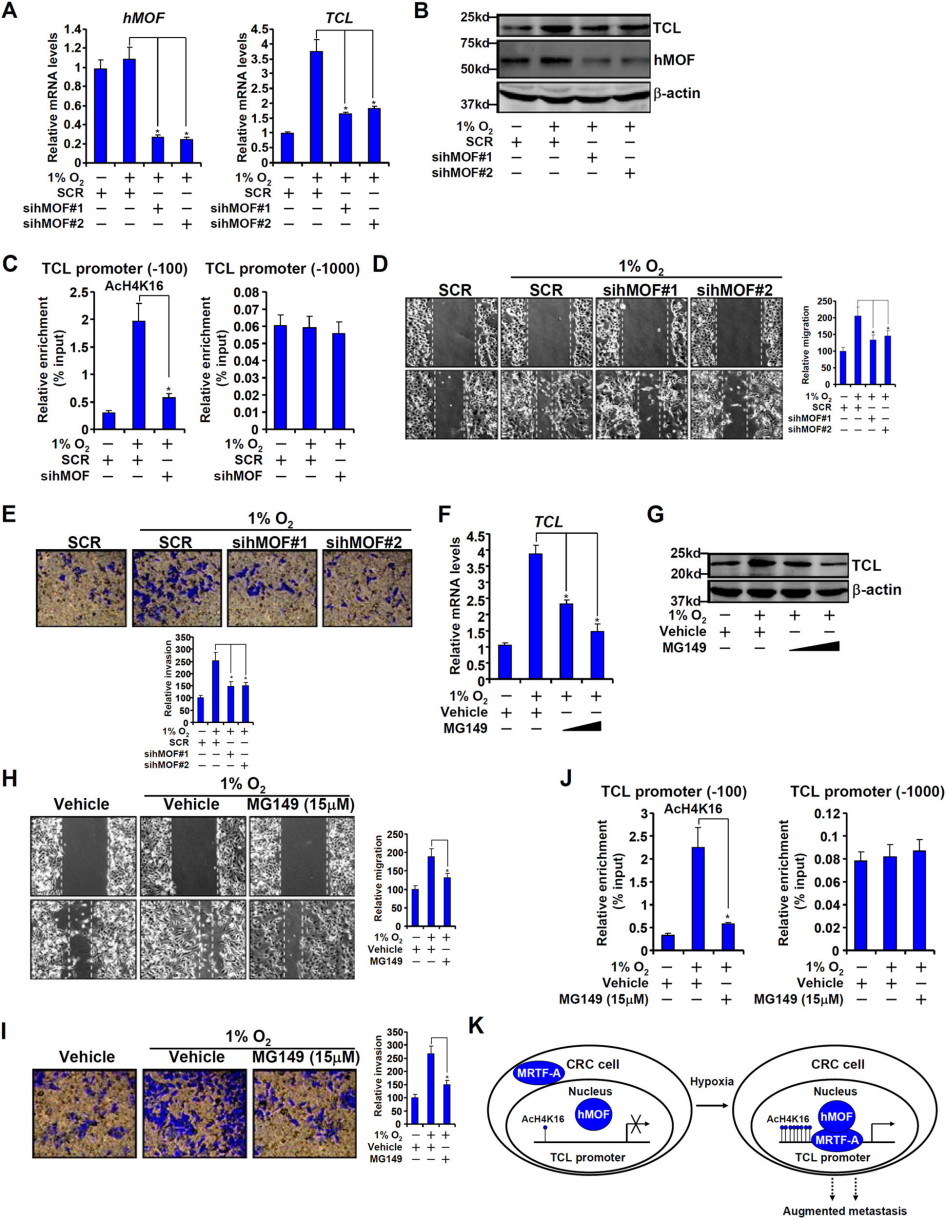
hMOF contributes to CRC malignancy by regulating TCL transcription. **a**–**e** HT-29 cells were transfected with siRNA targeting hMOF or scrambled siRNA (SCR) followed by treatment with 1% O_2_ for 48 h. Gene expression levels were examined by qPCR (**a**) and western (**b**). ChIP assay was performed with anti-acetyl H4K16 (**c**). Cell migration (**d**) and invasion (**e**) were measured by scratch wound healing assay and transwell assay, respectively. **f**–**j** HT-29 cells were treated with 1% O_2_ in the presence or absence of MG149 for 48 h. Gene expression levels were examined by qPCR (**f**) and western (**g**). ChIP assay was performed with anti-acetyl H4K16 (**h**). Cell migration (**i**) and invasion (**j**) were measured by scratch wound healing assay and transwell assay, respectively. Data represent averages of three independent experiments and error bars represent SEM. **k** A schematic model.

Two strategies were exploited to verify whether hMOF may be essential for hypoxia-induced TCL expression in CRC cells. First, siRNAs were used to knock down hMOF expression, which was accompanied by attenuation of hypoxia-induced TCL expression (Fig. 6a, b). ChIP assay showed that hMOF knockdown blocked the accumulation of H4K16 acetylation surrounding the TCL promoter following hypoxia stimulation (Fig. 6c). We next determined whether hMOF may be functionally relevant in CRC malignancy. Quantitative PCR showed that hMOF expression was elevated in highly malignant CRC specimens compared to those with a lesser malignant grade (Fig. 1c). Wound healing and transwell assays confirmed that hMOF depletion weakened migration (Fig. 6d) and invasion (Fig. 6e) of HT-29 cells exposed to hypoxia.

Alternatively, we used a small-molecule hMOF inhibitor MG149 to treat HT-29 cells. MG149 treatment not only repressed the induction of TCL expression by hypoxia (Fig. 6f, g) and reduced the accumulation of H4K16 acetylation surrounding the TCL promoter (Fig. 6j), but mitigated the aggressive migratory and invasive behaviors of hypoxia-treated HT-29 cells (Fig. 6h, i). Collectively, these data clearly demonstrate that hMOF contributes to CRC malignancy by regulating TCL transcription.

## Discussion

The past decade has witnessed the birth of cancer epigenomics along with great expansion in both scope and depth of our understanding with regard to the epigenetic regulation of cancer development and progression^22^. Here we present evidence to show that the histone H4K16 acetyltransferase hMOF, through its interaction with MRTF-A, activates the transcription of the small GTPase TCL to promote colorectal cancer cell migration and invasion (Fig. 6k). Mounting evidence suggests that hMOF may play a critical role in cancer biology. For instance, Jaganathan et al. observed that augmented proliferation of prostate cancer cells can be accounted for, at least in part, by hMOF potentiating NF-κB and androgen receptor (AR)-dependent transcription^23^. It has been reported by Chen et al. that low hMOF expression is associated with better prognosis in patients with non-small cell lung cancer^24^. Mechanistically, hMOF directly acetylates a non-histone transcription factor Nrf2 to up-regulate anti-drug genes. Alternatively, Zhao et al. have demonstrated that hMOF activates the transcription of Skp2, an E3 ubiquitin ligase that drives cell cycling, to promote NSCLC tumorigenesis^25^. Several independent studies have pointed to a role for hMOF in facilitating DNA repair in cancer cells to avoid growth arrest and apoptosis^26–28^. Consistent with our results as summarized here, Giampieri et al. have shown that hMOF, along with a panel of other genes, can be used to predict relapse in high-risk CRC patients^29^. Recently, genomewide gene expression analysis in mouse embryonic fibroblast (MEF) cells suggests that hMOF regulates the transcription of genes involved in stress response^30^, but it remains to be determined whether hMOF directly binds to the promoters to regulate transcription or whether hMOF regulates transcription through influencing the acetylation levels of H4K16 or non-histone factors. Future studies exploiting the ChIP-seq technique would provide more insights on the regulatory role of hMOF in CRC pathogenesis.

We show here that TCL promotes CRC cell migration and invasion (Fig. 2) and that TCL levels can be used as a predictor of CRC malignancies (Fig. 1). The mechanism whereby TCL regulates CRC metastasis remains an open question. Hypoxia can stimulate colorectal cancer cell migration and invasion by promoting angiogenesis^31^, epithelial-mesenchymal transition (EMT)^4^, and cancer stem cell (CSC) self-renewal^32^. It has been previously demonstrated that TCL blockade leads to failed angiogenesis during breast cancer metastasis^18^. We have previously shown^19^ that TCL is essential for hypoxia-induced endothelial-mesenchymal transition (EndMT), a pathophysiological process that shares many similar regulatory molecules with EMT, by regulating the activity of TWIST1 and SNAIL, two transcription factors with well-documented roles in promoting CRC metastasis^33^. There have also been indications that TCL might be involved in the regulation of stemness in different cells^34–36^. Additional investigations are warranted to sort out these lingering issues.

There is increasing evidence to suggest that MRTF-A plays a key role in carcinogenesis including lung cancer^37^, breast cancer^38^, and hepatocellular cancer^39,40^. Here we show that MRTF-A contributes to hypoxia-induced CRC cell migration and invasion by recruiting hMOF to activate TCL transcription. Of note, MRTF-A has been shown to interact with a plethora of epigenetic factors to promote carcinogenesis. For instance, an interaction between MRTF-A and the histone H3K4 methyltransferase complex COMPASS results in activation of MMP9 transcription and augmented migration/invasion of lung cancer cells^37^. Jehanno et al. have observed that a dynamic association between MRTF-A activity and H3K9 methylation status may account for breast cancer metastasis^41^. In addition, recruitment of histone acetyltransferase CBP/p300 by MRTF-A has been found to be a rate-limiting step in metastasis-related transcriptional events in cancer cells^38,42,43^. The chromatin remodeling protein BRG1 has been demonstrated to mediate MRTF-A-dependent trans-activation of MMP2 gene in ovarian cancer cells^44^. Crosstalk between the various MRTF-A-interacting epigenetic factors has been noted. BRG1, for example, is associated with both an H3K4 methyltransferase activity and an H3K9 demethylase activity likely by interacting with COMPASS^45,46^ and JMJD1A^47^, respectively. MRTF-A appears to play a brokering role central to the communications between these factors^45^. Whether hMOF can form a dialog with other epigenetic factors to cooperatively up-regulate TCL transcription and CRC cell migration/invasion awaits further investigation.

In summary, our data suggest that an MRTF-A-hMOF-TCL axis may contribute to colorectal cancer metastasis. It should be noted that these data are largely based on cell culture experiments and a rather small number of human specimens. Future studies should aim to validate our finding by using large cohorts of human data and by exploiting multiple clinically-relevant animal models. Small-molecule inhibitors targeting MRTF-A or hMOF have been found effective in the intervention of a host of diseases in animal models^48,49^. On other hand, specific TCL inhibitors are not available. Our findings therefore provide a strong rationale for the development of novel TCL-targeting reagents for the treatment of malignant colorectal cancers.

## Materials and methods

### Human colorectal cancer samples

All human studies were reviewed and approved by the intramural Nanjing Medical University Committee on Ethical Conduct of Studies with Human Subjects. Colorectal cancer tissues were collected, under informed consent, from surgical resection specimens of patients who had not undergone radiotherapy or chemotherapy in the Affiliated Hospital of Nantong University. Diagnoses of all cases were confirmed by histological examination. Tumor differentiation was graded by the Edmondson grading system. Samples were processed essentially as previously described^50,51^.

### Cell culture, plasmids, transient transfection, and reporter assay

Human colorectal cancer cells HT-29, HCT116, SW480, and Caco-2 have been previously described^52^. The cells were re-authenticated using a fingerprint method every 6 months in the laboratory. Where indicated, hypoxia (1% O_2_) was achieved by a mixture of ultra-high purity gases (5% CO_2_, 10% H_2_, 85% N_2_) in a 37 ^o^C incubator (Thermo Fisher). MRTF-A expression constructs^53,54^, hMOF expression constructs^48^, TCL expression constructs^12^, and TCL promoter-luciferase constructs^20^ have been previously described. For transient transfection, cells were plated in 12-well culture dishes (~60,000 cells/well). The next day, equal amounts (0.1 μg) of reporter construct and effector construct were transfected into each well. DNA content was normalized by the addition of an empty vector (pcDNA3). For monitoring transfection efficiency and for normalizing luciferase activity, 0.02 μg of GFP construct was transfected into each well. Raw luciferase activities were divided by both protein concentration and GFP fluorescence. Data are expressed as relative luciferase unit compared to the control group arbitrarily set as 1.

### Protein extraction, immunoprecipitation, and western blot

Whole-cell lysates were obtained by re-suspending cell pellets in RIPA buffer (50 mM Tris pH7.4, 150 mM NaCl, 1% Triton X-100) with freshly added protease inhibitor (Roche) as previously described^55–59^. Specific antibodies or pre-immune IgGs (P.I.I.) were added to and incubated with cell lysates overnight before being absorbed by Protein A/G-plus Agarose beads (Santa Cruz). Precipitated immune complex was released by boiling with 1X SDS electrophoresis sample buffer. Western blot analyses were performed with anti-TCL (Abcam, ab105311), anti-FLAG (Sigma, F3165), anti-HA (Invitrogen, 26183), anti-hMOF (Bethyl Laboratories, A300-992A), anti-MRTF-A (Santa Cruz, sc-32909), and anti-β-actin (Sigma, A2228) antibodies. All experiments were repeated three times.

### RNA isolation and real-time PCR

RNA was extracted with the RNeasy RNA isolation kit (Qiagen) as previously described^60–64^. On-membrane DNase digestion was performed during RNA extraction to eliminate contamination of genomic DNA. Reverse transcriptase reactions were performed using a SuperScript First-strand Synthesis System (Invitrogen). SYBRgreen real-time PCR reactions were performed on an ABI Prism 7500 system with the following primers: *TCL*, 5′-CGGCTGCAATGGACATGAG-3′ and 5′-GGCACGTATTCCTCTGGGAAG-3′; *MRTF-A*, 5′-ACCGTGACCAATAAGAATGC-3′ and 5′-CCGCTCTGAATGAGAATGTC-3′; *hMOF*, 5′- GAAGGAGCATGAGGCGATCA-3′ and 5′-TTTCGTAGTTCCCGATGTGGAT-3′. Ct values of target genes were normalized to the Ct values of a housekeekping control gene (18s, 5′-CGCGGTTCTATTTTGTTGGT-3′ and 5′-TCGTCTTCGAAACTCCGACT-3′) using the ΔΔCt method^65^ and expressed as relative mRNA expression levels compared to the control group which is arbitrarily set as 1. All experiments were performed in triplicate wells and repeated three times.

### Scratch-wound healing/migration assay

Cells were re-suspended in serum-free media. When the cells reached confluence, scratch wound was created by using a sterile micropipette tip. Cell migration was measured 24 h after the creation of the wound and calculated by Image Pro. Data were expressed as % migration compared to control arbitrarily set as 100%.

### Boyden chamber invasion assay

24-well inserts (Costar) with 10 μg/ml Matrigel (Sigma) were used for invasion assays. Cells were re-suspended in serum-free media and plated into the upper chamber with the lower chamber filled with complete media. Following exposure to indicated stimuli, the cells on the upper chamber were removed. Invaded cells were stained with 0.1% crystal violet and counted. Data were expressed as % invasion compared to control arbitrarily set as 100%.

### Chromatin immunoprecipitation (ChIP)

Chromatin Immunoprecipitation (ChIP) assays were performed essentially as described before^47,66–80^. In brief, chromatin in control and treated cells were cross-linked with 1% formaldehyde for 8 min at room temperature, and then sequentially washed with ice-cold phosphate-buffered saline, Solution I (10 mM HEPES, pH 7.5, 10 mM EDTA, 0.5 mM EGTA, 0.75% Triton X-100), and Solution II (10 mM HEPES, pH 7.5, 200 mM NaCl, 1 mM EDTA, 0.5 mM EGTA). Cells were then incubated in lysis buffer (150 mM NaCl, 25 mM Tris pH 7.5, 1% Triton X-100, 0.1% SDS, 0.5% deoxycholate) supplemented with protease inhibitor tablet and PMSF. DNA was fragmented into 200–500 bp pieces, verified by agarose electrophoresis, using a Branson 250 sonicator. Aliquots of lysates containing 200 μg of protein were used for each immunoprecipitation reaction with anti-acetyl H3 (Millipore, 06-599), anti-acetyl H4 (Millipore, 06-866), anti-acetyl H4K5 (Millipore, 07-327), anti-acetyl H4K8 (Millipore, 07-328), anti-acetyl H4K12 (Millipore, 07-595), anti-acetyl H4K16 (Millipore, 07-329), anti-hMOF (Bethyl Laboratories, A300-992A), anti-MRTF-A (Santa Cruz, sc-32909), or pre-immune IgG followed by adsorption to protein A/G PLUS-agarose beads (Santa Cruz Biotechnology). Precipitated DNA-protein complexes were washed sequentially with RIPA buffer (50 mM Tris, pH 8.0, 150 mM NaCl, 0.1% SDS, 0.5% deoxycholate, 1% Nonidet P-40, 1 mM EDTA), high salt buffer (50 mM Tris, pH 8.0, 500 mM NaCl, 0.1% SDS, 0.5% deoxycholate, 1% Nonidet P-40, 1 mM EDTA), LiCl buffer (50 mM Tris, pH 8.0, 250 mM LiCl, 0.1% SDS, 0.5% deoxycholate, 1% Nonidet P-40, 1 mM EDTA), and TE buffer (10 mM Tris, 1 mM EDTA pH 8.0). DNA-protein cross-link was reversed by heating the samples to 65 °C overnight. Proteins were digested with proteinase K (Sigma) at 37 °C for 2 h, and DNA was phenol/chloroform-extracted and precipitated by 100% ethanol. For re-ChIP, immune complexes were eluted with the elution buffer (1% SDS, 100 mM NaCO_3_), diluted with the re-ChIP buffer (1% Triton X-100, 2 mM EDTA, 150 mM NaCl, 20 mM Tris pH 8.1), and subject to immunoprecipitation with a second antibody of interest. Dried DNA was dissolved in 50 μl of deionized distilled water, and 5 μl was used for amplification by real-time PCR with the following primers: *TCL* proximal promoter, 5′-AGTGGGACCCCTAGTGTTTTC-3′ and 5′-AGGAAATCATGGGTTTCCTG-3′; *TCL* distal promoter, 5′-GGGTTCCTATAAATACGGACTGC-3′ and 5′-CTGGCACTGCACAAGAAGA-3′. A total of 10% of the starting material is also included as the input. Data are then normalized to the input and expressed as % recovery relative the input. All experiments were performed in triplicate wells and repeated three times.

### Statistical analysis

Sample sizes reflected the minimal number needed for statistical significance based on power analysis and prior experience. One-way ANOVA with post-hoc Scheffe analyses were performed using an SPSS package. Unless otherwise specified, *P* values smaller than 0.05 were considered statistically significant.

## Supplementary information

online supplementary figures
