# Supplementary material for: Epigenetic activation of the small GTPase TCL contributes to colorectal cancer cell migration and invasion
Source: Oncogenesis. 2020 Sep 30;9(9):86. doi: 10.1038/s41389-020-00269-9 (PMC7528090; doi:10.1038/s41389-020-00269-9)
Supplement: Supplementary file 1 — online supplementary figures [file 41389_2020_269_MOESM1_ESM.docx]

**Chen B *et al*: Epigenetic activation of the small GTPase TCL contributes to colorectal cancer cell migration and invasion**

**Online supplementary material**

**Fig.S1:** (**A, B**) HCT116 cells were exposed to 1% O_2_ and harvested at indicated time points. TCL expression levels were examined by qPCR and Western.

**Fig.S2:** (**A**) A constitutively active (CA) MRTF-A was transfected into HCT116 cells with or without siRNA targeting TCL. Cell migration was measured by scratch wound healing assay and quantified by Image Pro as described in Methods. (**B**) A constitutively active (CA) MRTF-A was transfected into HCT116 cells with or without siRNA targeting TCL. Cell invasion was measured by transwell assay and quantified by Image Pro as described in Methods. (**C**) HCT116 cells were transfected with siRNA targeting MRTF-A or scrambled siRNA (SCR) in the presence or absence of TCL followed by treatment with 1% O2 for 48h. Cell migration was measured by scratch wound healing assay and quantified by Image Pro as described in Methods. (**D**) HCT116 cells were transfected with siRNA targeting MRTF-A or scrambled siRNA (SCR) in the presence or absence of TCL followed by treatment with 1% O2 for 48h. Cell invasion was measured by transwell assay and quantified by Image Pro as described in Methods. (**E, F**) HCT116 cells were transfected with siRNA targeting MRTF-A or scrambled siRNA (SCR) followed by treatment with 1% O2 for 48h. Gene expression levels were examined by qPCR and Western.

**Fig.S3:** (**A**) SW480 cells and Caco-2 cells were transfected with siRNAs targeting MRTF-A, TCL, or scrambled siRNA (SCR). Cell migration was measured by scratch wound healing assay and quantified by Image Pro as described in Methods. (**B**) SW480 cells and Caco-2 cells were transfected with siRNAs targeting MRTF-A, TCL, or scrambled siRNA (SCR). Cell invasion was measured by transwell assay and quantified by Image Pro as described in Methods. (**C, D**) SW480 cells were transfected with siRNAs targeting MRTF-A or scrambled siRNA (SCR). Gene expression levels were examined by qPCR and Western. (**E, F**) Caco-2 cells were transfected with siRNAs targeting MRTF-A or scrambled siRNA (SCR). Gene expression levels were examined by qPCR and Western.

**Fig.S4:** HT-29 cells were transfected with siRNAs targeting MRTF-A or scrambled siRNA (SCR) followed by treatment with 1% O_2_. The cells were harvested at indicated time points and ChIP assays were performed with anti-RNA Pol II.

**Fig.S5:** SW480 cells and Caco-2 cells were transfected with siRNAs targeting MRTF-A or scrambled siRNA (SCR). ChIP assays were performed with anti-MRTF-A or anti-RNA Pol II.

**Fig.S6**: HCT116 cells were exposed to 1% O_2_ for 48h. Re-ChIP assay was performed with indicated antibodies.
